# Evolution of the Structure of EDPM Crosslinking Networks and Its Influence on the Rheological Properties of the Injection Molding Process

**DOI:** 10.3390/polym17182438

**Published:** 2025-09-09

**Authors:** Salvador Gomez-Jimenez, Carlos Guerrero Mendez, Daniela Lopez-Betancur, Antonio Robles-Guerrero, David Navarro-Solis, Luis Silva-Acosta, Enrique A. Lopez-Baltazar, Jennifer Ortiz-Letechipia, Ada Rebeca Contreras-Rodríguez

**Affiliations:** 1 Engineering Academic Unit, Autonomous University of Zacatecas, Avenue López Velarde 801, Zacatecas 98000, Mexico; danielalopez106@uaz.edu.mx (D.L.-B.); aroblesp@uaz.edu.mx (A.R.-G.); david.navarro@uaz.edu.mx (D.N.-S.); ealopezb@uaz.edu.mx (E.A.L.-B.); 2 Academic Unit of Science and Technology of Light and Matter, Autonomous University of Zacatecas, Campus Siglo XXI, Zacatecas 98160, Mexico; guerrero_mendez@uaz.edu.mx (C.G.M.); luis.silvaa@uaz.edu.mx (L.S.-A.); 3 Chemical Sciences Academic Unit, Autonomous University of Zacatecas, Avenue López Velarde 801, Zacatecas 98000, Mexico; jenniol@uaz.edu.mx

**Keywords:** EPDM, rheological properties, crosslinking mechanisms, molecular dynamics simulations, injection molding process, mean square displacement

## Abstract

The rubber industry is evolving by incorporating innovative tools to improve production processes. A proper manufacturing process determines the behavior and service life of the resulting products. In this research, molecular dynamics simulations were used to study the effect of temperature in the cured structure on the resulting mechanical properties of EPDM. The results of the simulations at different temperatures of the crosslinked ethylene–propylene–diene monomer (EPDM) were then compared in terms of the radius of gyration, free volume, root mean square displacement, stress curves, viscosity, and gel point. Then, using the superposition principle, viscosity and tensile stress were evaluated. The molecular dynamics superposition results could reasonably predict the mechanical behavior of EPDM during and after the injection process. The results provide new insights into the molecular-level crosslinking mechanisms of amorphous polymers and their influence on mechanical behavior, which facilitates the design of the injection process for rubber component applications. The results show an increase in viscosity and a decrease in the critical gel point with increasing temperature. The hardness tests performed on an automotive component demonstrate that this has an impact on the resulting properties.

## 1. Introduction

Elastomers are valued in industrial applications for their notable properties, such as elasticity, chemical stability and durability. Sealing systems rely heavily on components fabricated from synthetic elastomers. Ethylene–propylene–diene monomer (EPDM) rubber is chemically stable; this type of elastomeric polymer is highly resilient and resistant to chemical agents. However, the fatigue strength and service life of EPDM components are significantly influenced by operational factors, including temperature conditions and the number of mechanical load cycles [1,2,3].

The manufacturing sector is experiencing a revolution (Industry 4.0), motivated by the demand to rapidly develop products that integrate automated, efficient, and sustainable technologies. In recent years, the rubber-component manufacturing industry has adopted sustainability and carbon footprint reduction policies; to fulfill these goals, the integration of virtual prototyping testing to develop high-quality, eco-friendly products has been implemented as sustainable practice in design and manufacturing [4,5,6].

The validity of the numerical simulations implemented depends on the accuracy of the correlation between the results obtained and the mechanical properties of the rubber material. Several analytical models have been proposed to represent the mechanical behavior of rubber-like materials. The molecular structure of elastomers enables these materials to sustain large elastic deformations under tensile loading, exhibiting characteristic nonlinear stress–strain behaviour. The complex mechanical response of crosslinked rubbery materials presents significant research challenges, particularly in characterizing how crosslinking and temperature interact during the curing process [7,8].

Different theories have been proposed to evaluate the effect of temperature on the crosslinking and viscoelastic connection, ranging from chain breaking in the amorphous structure to the sliding of molecules and to the formation of more complex composite structures. Most experimental techniques must infer molecular mechanisms from measurements. Theoretical tools, including molecular information, could complement these interpretations. Trying to understand the stability of molecular structures at an atomistic level is something that theoretical and experimental scientists are working on. The intention is to develop models capable of reproducing experimental observations from molecular dynamics simulation results. Advances in computing power have made it possible to develop simulation models that come closer and closer to reality and help us to understand what is happening at the nanoscopic level [9].

For the crosslinking process during curing, because of the degree of complexity, it is evident that no current model can accurately and effectively predict the temperature and crosslink density, dependent on viscoelastic response of an elastomer in a thermodynamically consistent and numerically sturdy manner, due to the strong non-linearity [10].

The crosslinking density significantly impacts the mechanical performance of elastomers. Viscoelastic properties change drastically with increasing crosslinking concentration. The temperature gradient during vulcanization influences the density and distribution of the crosslinks and, therefore, the mechanical properties that result from the curing process [11].

The viscoelasticity of crosslinked polymers is influenced by both chain morphology and length. Therefore, it is important to determine the dynamics of the system. Theoretically, in the amorphous structure, the dynamics are locally constrained by crosslinking and entanglement of the chains. The configurational freedom of mobility is influenced by friction and intermolecular coordination [12]. The viscoelasticity of rubber is directly related to the of chemical and physical crosslinks during the curing process, with key parameters like chain length, crosslink density, and entanglements, which dictate the dynamics and stored elastic entropy [13].

Several studies have been conducted using molecular dynamics simulations of polymer properties to investigate the kinetic properties of EPDM in terms of free volume and crosslink density. The crosslink density is a key factor that shapes mechanical behavior. Viscoelasticity parameters are constitutionally linked to intrinsic material properties, such as network structures and intermolecular forces [14].

Molecular dynamics simulation is a computational tool that facilitates the analysis of the behavior or evolution of a system over time. This technique provides a robust method for the generation of non-equilibrium ensembles and the analysis of dynamical events at atomistic scales by making the trajectories of a system composed of a specific number of particles, solved for very short times, with specifications of an interatomic interaction potential, initial conditions and boundary constraints [15].

Wang et al. [16] utilized molecular dynamics simulations to envision the fact that crosslinking within the side chains increases stiffness and reduces diffusion properties. These results provide a deeper insight into the microscopic origins of EPDM mechanical behavior. While Wang et al. [17] use time–temperature superposition and varying strain rates in molecular dynamics simulations, the data collected allow modeling of the rheological properties of EPDM.

A significant advance in the modeling of crosslinked polymers emerged with the development of the multistep methodology; Varshey et al. [18] proposed a step-by-step method that involves constantly increasing the cut-off reaction radius in a relatively large crosslinked model, in order to simulate the crosslinking reaction. Overall, to simulate the EPDM crosslinking process by implementing some step-by-step or multistep algorithm, the cut-off reaction radius should be modified periodically, as this is the key to simulating the EPDM crosslinking process; the most prominent works in this regard, to mention a few, are Papanikolaou et al., Wang et al., and van Duin et al. [16,19,20].

This research approaches the study of the conformation of a crosslinked polymeric system, considering the crosslink density in the amorphous structure of EPDM using molecular dynamics modeling. In this study, the methodology proposed considers the dynamics of the polymeric chains in the amorphous structure for the viscoelastic characterization of EPDM, using trajectory analysis.

## 2. Materials and Methods

### 2.1. Topology of the System Subsection

**Figure 1:**
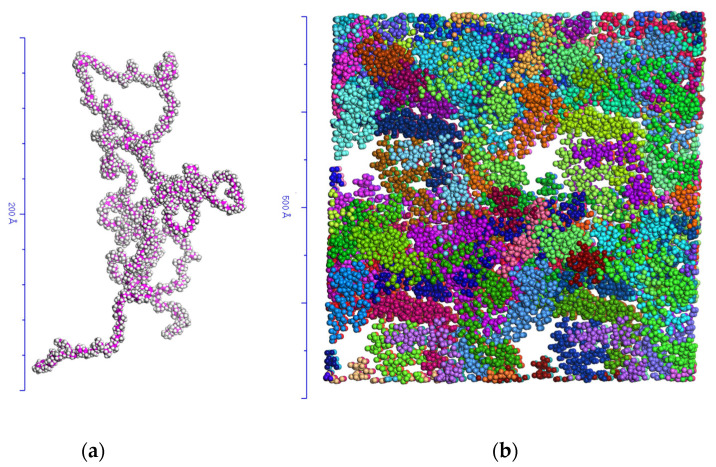
(**a**) Polymer chain with 500 monomers. (**b**) Simulation box with 10 polymer chains; each color represents a chain.

The simulations in molecular dynamics (MD) assume a configuration of non-overlapping of all the chains of the amorphous EPDM polymer. The procedure to define the topology of the system was to generate the repeating unit (monomer) and then build the polymer chain with the monomers (Figure 1a). The resulting topology was optimized (pre-equilibrium) in 10,000 steps at each stage. The simulation box consists of 10 chains with 500 monomers each, randomly distributed (Figure 1b). Three-dimensional periodic boundary conditions are applied to eliminate surface effects.

This configuration allows for modeling of chain flexibility and entanglement. Short chains allow simulation of properties relevant to rubber injection, such as viscosity and chain dynamics, while keeping computational costs low [19,21,22,23].

### 2.2. Interatomic Potential Parameterization

The interatomic potential implemented in this research was calculated using the condensed-phase optimized molecular potentials for atomistic simulation studies (COMPASS). The COMPASS force field employs a simplified representation of the Coulomb and van der Waals force interactions [24,25].

Recent research has demonstrated the effectiveness of using the COMPASS force field for amorphous polymers and elastomers, which is a significant finding. In their study, Saha et al. [26] employed COMPASS to visualize the macromolecular chains of various flexible amorphous polymers. They evaluated the diffusive characteristics of these chains using the mean square displacement, a method that describes the mobility of polymer chains. Guo et al. [27] studied the compatibility between elastomers and resins, using a combination of experimental results and molecular dynamics simulation. The MD simulation process involved calculations performed using the COMPASS force field, which yielded consistent results and led to the development of a novel and efficient method for creating new resins. The effect of accelerators on the vulcanization of styrene-ethylene–butylene–styrene block copolymer (SEBS) rubber was investigated by Yang et al. [28]. They successfully employed molecular dynamics simulations using the COMPASS force field; meanwhile, Xiang et al. [29] used the COMPASS parameterized force field to predict the macroscopic properties of polydimethylsiloxane (PDMS) from its microscopic structure, using molecular simulations. The results showed reasonable agreement with experimental data in terms of PDMS’s thermodynamic, structural, and vibrational properties.

The accuracy, robustness, and adaptability required for complex polymeric elastomer systems, which have many industrial process applications such as rubber injection molding, are provided by the COMPASS force field. COMPASS is optimized for condensed-phase systems, making it ideal for simulating elastomeric polymers, which exhibit complex interactions in the solid or gel state. It incorporates energy terms that accurately depict intramolecular interactions (e.g., bonds, angles, and torsions) and intermolecular interactions (e.g., van der Waals and electrostatic forces) in dense systems [30,31,32].

The van der Waals interactions maintain atoms of opposite charge with a specific separation distance between each other. For electrostatic and van der Waals interactions, summation was performed using the Ewald and atom-based algorithms, respectively [24,33].

The interaction potential for a polymer chain system includes valence terms parameterized by t internal bond coordinates (b), angle (θ), torsion angle (φ), and out-of-plane angle (χ). Vibrational frequencies and structural variations arising from conformational changes are interlinked through cross-coupling mechanisms. These terms include combinations of two or three internal coordinates considering the bond angle potential ($E_{ba}$), the in-plane dihedral rotation potential ($E_{dh}$), the out-of-plane vibrational potential ($E_{inv}$), and the cross term ($E_{cross}$). Non-bonding interactions are described by Lennard-Jones 9-6 potential for the van der Waals term ($E_{vdW}$) and a Coulomb function for an electrostatic interaction ($E_{coul}$). The functional terms in COMPASS are given by the Equation (1)

(1) $$E_{total}=E_{vdW}+E_{coul}+E_{ba}+E_{dh}+E_{inv}+E_{cross}$$

where $E_{total}$ is the total potential energy of the system, $E_{vdW}$ is the potential energy due to intermolecular forces, $E_{coul}$ is the Coulomb electrostatic potential, $E_{ba}$ is the bond angle potential, $E_{dh}$ is the dihedral rotation potential within the molecule, $E_{inv}$ is the off-plane vibrational potential, and $E_{cross}$ is the cross term.

The potential energy due to the intermolecular forces $E_{vdW}$ can be calculated by Equation (2):

(2) $$E_{vdW}=\sum_{ij}\varepsilon_{ij}\left[{2\left(\frac{\sigma_{ij}}{r_{ij}}\right)}^{9}-{3\left(\frac{\sigma_{ij}}{r_{ij}}\right)}^{6}\right]\;\;$$

where the subscripts ij in the summations represent the potential energy interactions, $\varepsilon_{ij}$ represents the potential energy parameter, $\sigma_{ij}$ represents the finite distance when the potential energy between molecules becomes zero, and $r_{ij}$ denotes cut-off distance between atoms i and j.

$E_{coul}$ is the Coulomb electrostatic potential, and can be expressed by Equation (3) below:

(3) $$E_{coul}=\sum_{ij}\frac{q_{i}q_{j}}{r_{ij}}\;$$

$q_{i}$ and $q_{j}$ are the fixed partial charges between atom i and j within the same molecule.

In the next Equation (4), where $E_{ba}$ is the bond angle potential, θ represents the energy associated with the angle, the value $\theta_{0}$ represents equilibrium, and k, $k_{1}$, $k_{2}$, and $\;k_{3}$ are force constants.

(4) $$E_{ba}=\sum_{\theta }\left[k_{2}{\left(\theta -\theta_{0}\right)}^{2}+k_{3}{\left(\theta -\theta_{0}\right)}^{3}+k_{4}{\left(\theta -\theta_{0}\right)}^{4}\right]\;$$

The intermolecular dihedral rotation potential $E_{dh}$ is defined by the next Equation (5), where ϕ represents the torsional angle.

(5) $$E_{dh}=\sum_{\phi }\left[k_{1}\left(1-cos\phi \right)+k_{2}\left(1-\cos2\phi \right)+k_{3}\left(1-\cos3\phi \right)\right]\;$$

The out-of-plane vibrational potential $E_{inv}$ is represented by Equation (6), where Wilson out-of-plane χ internal coordinates are the following:

(6) $$E_{inv}=\sum_{\chi }k_{x}\chi^{2}\;$$

Equation (7) $E_{cross}$ includes the cross-coupling terms to calculate the vibrational frequencies and structural variations associated with conformational changes.

(7) $$\begin{array}{c} E_{cross}=\sum_{b,b’}k\left(b-b_{0}\right)\left(b’-{b’}_{0}\right)+\sum_{b,\theta }k\left(b-b_{0}\right)\left(\theta -\theta_{0}\right)\\ +\sum_{b,\phi }k\left(b-b_{0}\right)\left(k_{1}cos\phi +k_{2}\cos2\phi +k_{3}\cos3\phi \right)\\ \begin{array}{c} +\sum_{\theta ,\phi }k\left(\theta -\theta_{0}\right)\left(k_{1}cos\phi +k_{2}\cos2\phi +k_{3}\cos3\phi \right)\\ +\sum_{\theta ,\theta ’}k\left(\theta -\theta_{0}\right)\left(\theta ’-{\theta ’}_{0}\right)+\sum_{\theta ,\theta ’,\phi }k\left(\theta -\theta_{0}\right)\left(\theta ’-{\theta ’}_{0}\right)cos\phi \end{array} \end{array}$$

The mean square displacement (MSD) is the parameter associated with the diffusion or mobility of molecules, and is related to the viscosity and relaxation of the polymer. To compare the kinetic properties of EPDM composed of different crosslink densities, the MSD of the particles is calculated during the MD simulation.

(8) $$MSD\equiv \langle {\left|x_{i}\left(t\right)-x_{i}(0)\right|}^{2}\rangle$$

where $x_{i}(0)$ and $x_{i}\left(t\right)$ are the positions of particle i at the initial time and time t, respectively; the angle brackets ⟨...⟩ indicate the average overall possible time.

### 2.3. Crosslinking Characteristics

**Figure 2:**
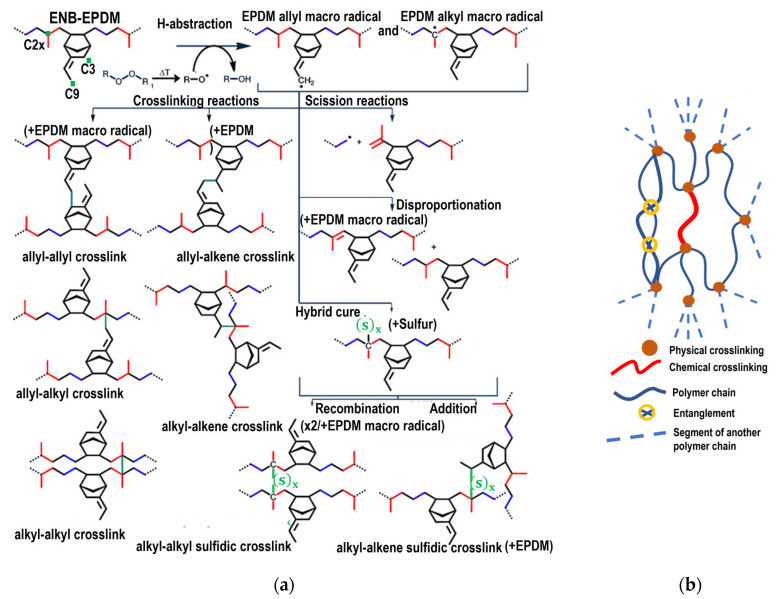
(**a**) Types of crosslinking that are possible during the curing of EPDM. (**b**) Schematic representation of the molecular structure of a cured elastomer (sulfur vulcanizing agent).

The chemical crosslinking process of EPDM is explained by taking as reference the investigations of Wang et al. [16,33] to model the process with a third 5-ethylidene-2-norbornene (ENB) monomer. van Duin et al. [20] and Papanikolaou et al. [19] showed that peroxide free radicals preferentially abstract the tertiary hydrogen (C2x) of the main chain and the hydrogens at the C3 and C9 positions of ENB, in agreement with Papanikolaou et al. The positions are shown schematically in Figure 2a. In EPDM, the main-chain-to-side-chain ratio is approximately 9:1. Zachary et al. [34] demonstrated that the monomer reaction rates were highly selective, with C3 exhibiting 90% reactivity compared to just 10% for C9. Crosslinking predominantly occurs between the C2x carbon of the main chain and the C3 carbon of side chains. Chemical crosslinking with the C9 atom was omitted because its reaction rate is quite low compared to the previous ones.

Physical crosslinking is also driven by weak interactions. These interactions arise from electrostatic interactions and van der Waals forces, which influence polymer gelation. In elastomers, physical crosslinking plays a critical role in determining mechanical properties and enhancing material stability. Figure 2b shows physical crosslinking.

### 2.4. Procedure for Molecular Dynamics Simulation

**Figure 3:**
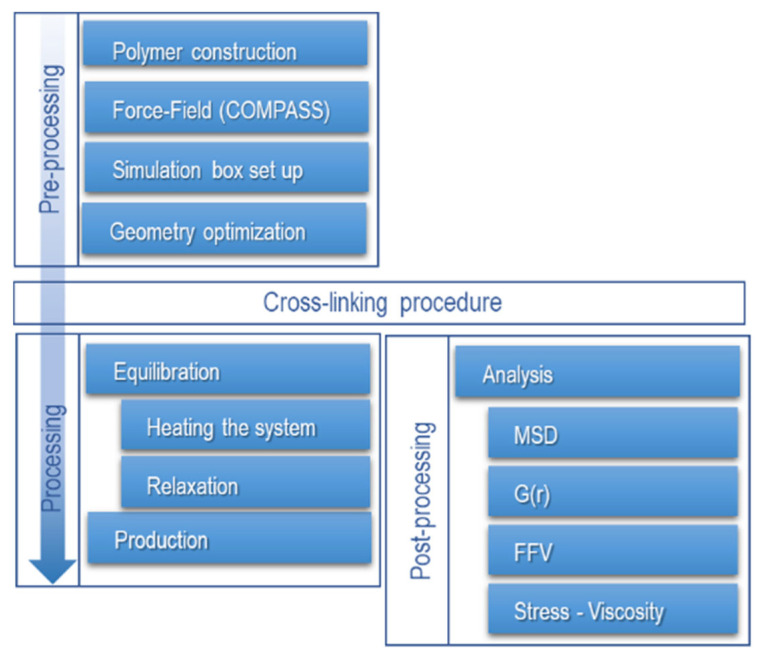
Procedure for molecular dynamics simulation. MSD is the mean square displacement, G(r) is the radial distribution function, and FFV is the fractional free volume.

Equilibration protocols are not fully standardized; although there is no general methodology for equilibrating MD systems, there are guidelines to follow. The proposed methodology in this study consists of equilibrating the system into two stages with four frames (replicas), where each frame’s topology is individually optimized during pre-equilibration (Figure 3).

Three-dimensional periodic boundary conditions are applied to the simulation box to minimize surface effects during dynamic crosslinking.

The motion equations were solved with the algorithm of Verlet velocity, and the integration time interval was set at 1 femtoseconds (fs); in all cases, for smaller time steps tested, no meaningful difference in the properties of interest was found [24]. The Boltzmann distribution was used to randomly generate the initial velocities [35,36]. The simulation temperatures were set in the range of 428 to 478 K, with increments of 10; control was carried out using the Andersen method [37]. A linear change was applied to the positions of the atoms to obtain a shape change.

The generated frames are equilibrated by varying the conditions to bring them to a stable energy state, defining scenarios with variables such as temperature, pressure, energy, and volume.

Simulated annealing is performed in the first equilibrium step with a constant energy and volume (NVE) assembly, which involves simulation of a system based on constant number of particles (N), constant volume (V), and constant energy (E); the sum of kinetic (KE) and potential energy (PE) is conserved, and T and P are unregulated in a temperature range of 350 to 500 K with 100 cycles and 10 ramps per cycle (temperature change of 15 K per ramp), and time step 1 fs, with 200,000 steps.

In the second step, the system was relaxed under canonical ensemble conditions (NVT: constant number of particles, volume, and temperature) using the Andersen thermostat to maintain temperature control, which typically adds a degree of freedom to the conserved Hamiltonian, and P is unregulated. On the obtained trajectories, to equilibrate the pressure variation, simulations were performed with the constant number of particles, pressure, and temperature (NPT) assembly at 100 MPa. To equilibrate the size of the simulation box and to obtain the correct system density for production, both simulations were performed with 20 nanoseconds (ns) and with a time step of 1 fs, and the process was repeated in the temperature range from 428 K to 478 K with 10 K steps.

### 2.5. Crosslinking Procedure

In the rubber injection industry, EPDM undergoes a vulcanization process to form a three-dimensional network of crosslinks, usually using sulfur or peroxides, which gives it its mechanical properties (e.g., elasticity and tear resistance). In practice, however, achieving complete crosslinking (100%) is difficult, due to kinetic limitations, mixture heterogeneity, and steric restrictions. A crosslinking degree of 90% is close to the maximum that vulcanized rubber compounds, such as EPDM, can achieve experimentally [38,39].

The crosslinking methodology follows the approach established by Papanikolaou et al. and adopted by Wang et al. [16,19]. Simulations involved calculating all potential crosslinks by evaluating distances between reactive atoms within a predefined cut-off of 0.4 nm and 1 nm and a crosslinking limit of 90%. The crosslinking kinetics were modeled as both random and periodic, generating covalent bonding terms accordingly. To prevent missing atoms, new bonds were activated by iteratively adjusting equilibrium distances and force coefficients. The lower boundary distance was incremented by 0.025 nm per iteration, with continuous checks on interatomic distances. This iterative process continued until the reactive pairs were exhausted within the cut-off distance and the crosslinking limit was achieved.

**Figure 4:**
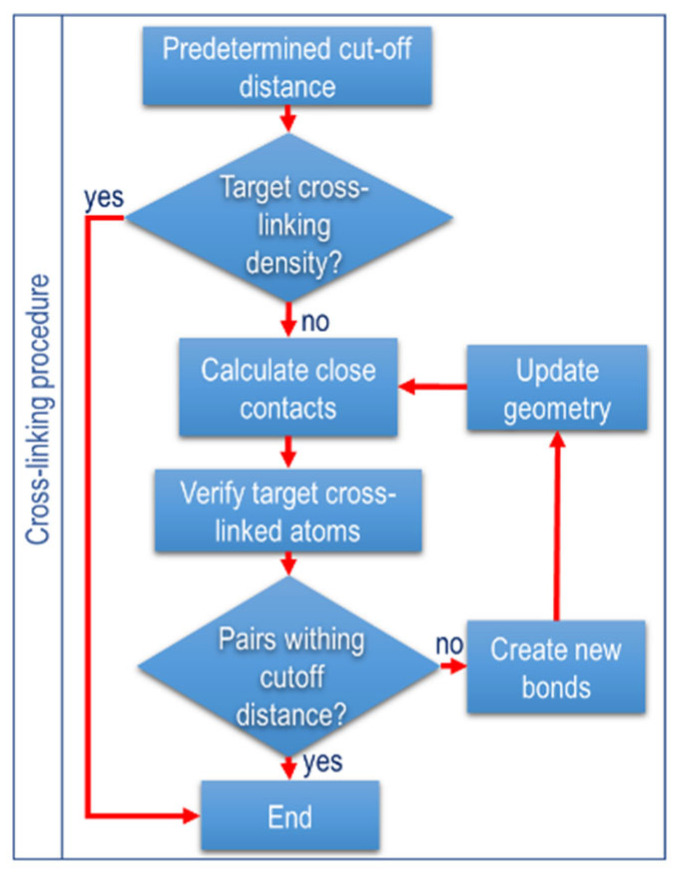
Procedure for molecular dynamics simulation as a flowchart of the crosslinking procedure.

After the initial models were sufficiently equilibrated to simulate the EPDM curing process, a step-by-step algorithm was employed to model the process (see Figure 4). Step 1: distances between reactive atoms are measured, and crosslinks are formed between pairs within a set cutoff distance. Step 2: covalent bonds are formed from these pairs, and the hydrogen atoms are removed from the newly formed bonds. Step 3: the new bonds are gradually activated by adjusting the equilibrium distances of the bonds and the force coefficients. This prevents the atoms from escaping the simulation box due to large atomic forces. Step 4: an NPT simulation is conducted, and the simulation box is stabilized. Then, distances between reactive atoms are rechecked. Step 5: the process repeats until no reactive pairs remain within the cutoff distance, reaching the crosslinking limit.

### 2.6. Parameters for the Study of Molecular Dynamics

Mean square displacement (MSD) in molecular dynamics is calculated using the Einstein formula, which defines the MSD as an ensemble average over time for the particles. The standard equation is

(9) $$MSD\left(t\right)=\langle {\left|r\left(t\right)-r\left(0\right)\right|}^{2}\rangle$$

The radius of gyration $\left(R_{g}\right)$ measures the average effective size of a polymer’s chains; in this case, it is used to quantify the degree of temperature-induced folding. Flexibility in molecular dynamic systems is characterized by the radius of gyration; a small value indicates that the polymer is relatively compact, meaning that, along the path, the polymer is folded most of the time. Thus, we can measure the radius of the gyration distribution of a polymer to characterize the folding patterns and analyze the conformational behavior.

(10) $$R_{g}=\sqrt{\frac{1}{N}\sum_{i=1}^{N}\langle s_{i}^{2}\rangle }$$

where $s_{i}^{2}$ is the quadratic modulus of the vector defined between the center of mass of the chain and the position occupied by backbone i in an instantaneous conformation of the polymer.

The end-to-end vector is the vector pointing from one end of a polymer to the other. For a discrete representation, where monomer units are mapped as points in space, their relative positions are described by translation vectors $\vec{r_{i}}$. The end-to-end vector is then computed as the vector sum.

(11) $${\vec{R}}_{n-1}=\sum_{i=1}^{n-1}{\vec{r}}_{i}$$

Radial distribution function g(r) describes the density variation as a function of distance measured from a reference particle. It measures the probability of finding a particle at a distance r from another marked particle. The density depends on the conformational structure of the polymer chains.

(12) $$g\left(r\right)=\frac{dn_{r}}{\rho dV_{r}};\;\;dV_{r}\approx 4\pi r^{2}dr$$

where ρ is bulk density, $dn_{r}$ is a function that computes the number of particles within a shell of thickness dr, and $dV_{r}$ is the spherical shell volume, with the approximation holding for small-shell thicknesses.

### 2.7. Equipment and Materials for Hardness Test

For the injection molding tests in this project, Elastomer Solutions GmbH provided the synthetic ethylene–propylene–diene monomer (EPDM) elastomer manufactured by Dow. The exact formula is confidential and proprietary information. The main components are listed in Table 1.

**Figure 5:**
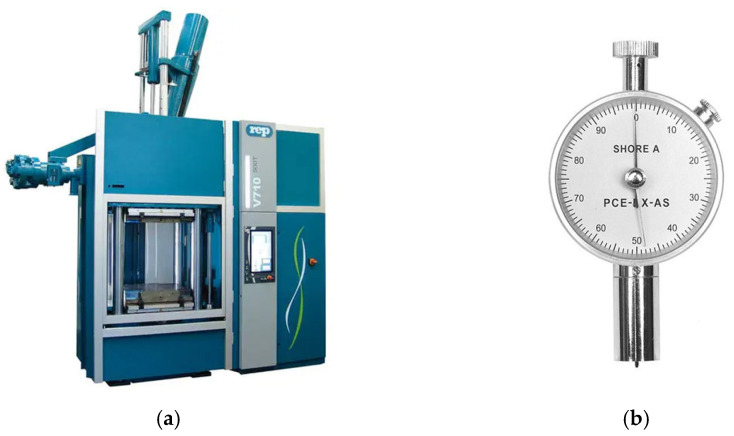
(**a**) Vertical injection machine REP International V710-Y2000. (**b**) PCE-DX-AS durometer.

Injection testing equipment: REP International V710-Y2000 vertical injection machine, G10 press from the Performance range, for rubber injection applications with precise process control and injection temperature, maximum injection capacity of 2000 cm^3^, and maximum injection pressure of 150 MPa. The heating plate dimensions are 630 mm × 800 mm, with a maximum mold plate surface size of 800 mm × 800 mm and a clamping force of 5100 kN, driven by hydraulic power. Width 3031 mm × depth 1746 mm height 4577 mm. Incorporates extended connectivity features (Industry 4.0, see Figure 5a).

The PCE-DX-AS durometer is a hardness testing instrument used to measure the Shore A hardness of rubber and soft rubbers. It consists of a single piece, featuring a measuring head and a 360° anti-glare dial for reading the hardness value. It includes a drag needle that remains at the maximum measurement point for reading the peak value, which must be reset to zero after each measurement (see Figure 5b). The measurement range is 0 to 100 Shore A units. Technical characteristics: weight: 160 g; pressure force: 0–12.5 N; penetration body: 35° (according to Shore A); dial diameter: 5.757 mm; total length: 110 mm; recommended measurement range: 10–90 Shore A (accuracy decreases outside this range); error limit: ±2; scale division: 1; drag needle designed to comply with DIN 53505, ASTM D2240, ISO/R868, JIS R7215, GB/T531-99, GB2411-80, HG/T2489-93 and JJG304-2003.

## 3. Results and Discussion

### 3.1. Mean Square Displacement (MSD)

At the molecular scale, vulcanization is defined by the formation of a crosslinking chain and polymer entanglement, resulting in significant mechanical property changes observed macroscopically as gel-point transitions. The 428–478 K range corresponds to typical processing temperatures (155–205 °C) for injection molding, where EPDM exhibits rubbery behavior, facilitating the simulation of chain mobility (e.g., mean squared displacement, radius of gyration) and crosslinking dynamics.

At elevated temperatures, the polymer network reaches a state of crosslinking saturation. This affects the resulting viscoelastic properties of the elastomer. Analysis of the mean square displacement (MSD) trajectories indicates that the mobility of EPDM molecules decreases as temperature rises. This phenomenon, however, is due to the combined effect of two key factors: restricted chain movement due to crosslinking, and the strong interactions at the bond interface.

**Figure 6:**
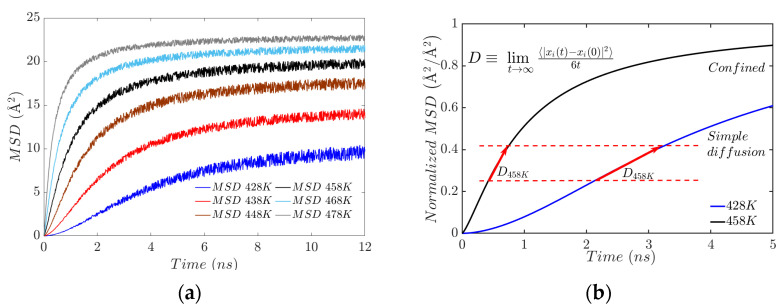
(**a**) Mean square displacement results. (**b**) Slope for mean square displacement normalized.

The increase in crosslinking, as well as the appearance of electrostatic interaction forces, means that the longer chains begin to intertwine with each other, leading to the rigidity of the network, which constrains it geometrically. In Figure 6a, this can be seen in the mean square displacement (MSD) trajectories: as the slope changes, the movement (diffusion) of the polymer chains is constricted. The confinement (Figure 6b) results in the coexistence of a crosslinked amorphous structure that forms a gel phase. The formation of crosslinks is associated with the system’s rigidity, manifested by a progressive increase in viscosity. To understand how the mobility or diffusion of the chains has changed, the behavior is plotted at the lowest (428 K) and intermediate (458 K) temperatures of the simulations. The blue line shows a quasi-linear slope, indicating slow but progressive diffusion. In contrast, the black line shows a non-linear slope that tends towards dynamic arrest.

The plots (Figure 6a) show that higher temperatures increase kinetic energy and diffusion, resulting in greater chain mobility. For example, the MSD reaches a value of 2.46 ${Å}^{2}$ at 2 ns and a temperature of 428 K, whereas at 478 K, the value is around 20.53 ${Å}^{2}$, which correlates with greater fluidity in the injection process.

When the MSD stabilizes, this suggests confinement due to the number of crosslinks needed to reach the gelation point. Normalizing the MSD values (dividing the MSD at time t by the maximum MSD reached; see Figure 6b) is analogous to the cure fraction proposed by Sun and Isayev [40], adopted by Milani et al. [41], and revisited by Gomez et al. [42]. At a temperature of 458 K, the cure fraction reaches approximately 90% at 5 s, whereas at 428 K it is only 60%.

### 3.2. Radius of Gyration (Rg)

**Figure 7:**
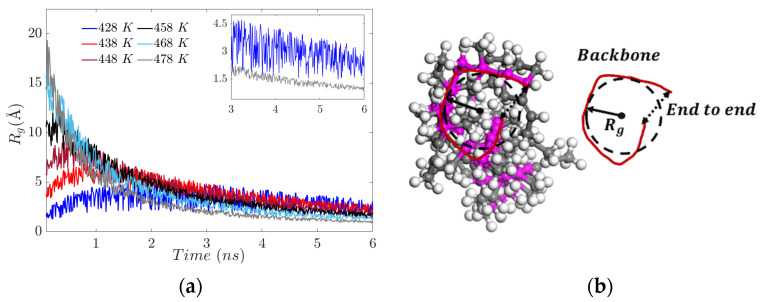
(**a**) Radius of gyration for different temperatures. (**b**) Diagram of the radius of gyration, mass distribution and end-to-end in the backbone.

The evolution of the radius of gyration in the polymer chains is linked with the elasticity (flexibility) of the resulting system, manifested by the progressive increase in stiffness. At the temperature of 428 K, there is greater flexibility in the main chain, which is expected to facilitate crosslink formation. In contrast, at 478 K, agglomeration of side chains is formed (see Figure 7a), while the main chain undergoes no significant conformational changes, due to limited free volume availability.

### 3.3. The End-to-End Vector

In the study of polymers, the measure of the distance end-to-end of the main chain indicates its degree of flexibility. The more flexible it is, the greater the probability of branching increasing. This measurement also shows a possible conformational change in the backbone or an aggregation of the side chains.

**Figure 8:**
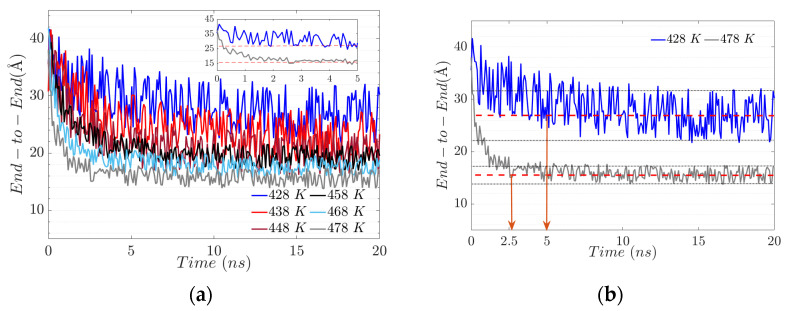
(**a**) End-to-end configuration at different temperatures. (**b**) Evolution of main chain end-to-end distance with maximum and minimum temperatures.

The end-to-end distance decreases by about 25% for the system at 428 K; this decrease occurs in the first 5 ns (Figure 8a). After that, the system does not show more than 9% variation. This shows the great flexibility of the main chain during the first 5 ns. In addition, when the system exceeds 5 ns, the flexibility tends to decrease by 46%, concerning the initial configuration. In the case of the system with a temperature of 478 K, the distance end-to-end during the first 2.5 ns decreases drastically by 42%, and, subsequently, the fluctuations stabilize in a range of 8%. The final ratio concerning the initial conformation is 68% lower, which indicates higher rigidity than the systems with lower temperatures (Figure 8b).

### 3.4. Radial Distribution Function (g(r))

**Figure 9:**
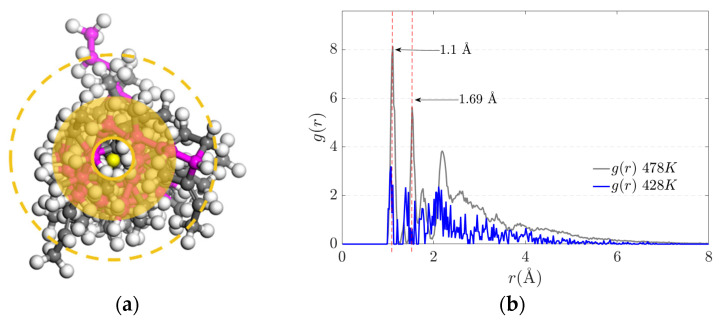
(**a**) Radial Distribution Schematic (**b**) Radial distribution function (or pair correlation function).

The radial distribution function denoted by *g*(*r*) (Figure 9a) defines the probability of finding a certain distance between two atoms of different polymeric chains where there is a physical connection by electrostatic forces. Figure 9b represents the radial distribution function where the electrostatic equilibrium distance (physical crosslink) is 1.1 Angstrom. The value of the radial function increases with temperature.

### 3.5. Free Volume

The free volume was determined by generating an atomic volume surface (the Connolly surface). The periodic box was meshed with square cells of 0.11 Å spacing, and all grids were scanned with a probe of radius 1.1 Å. Grid cells, where the probe occupied over 50% of the space were classified as accessible, while those predominantly occupied by atoms were labeled as occupied. In polymeric systems, free volume emerges when consecutive grid cells remain unoccupied. Therefore, the fractional free volume (FFV) corresponds to the ratio of this unoccupied volume (free volume) to the total volume of the system.

**Figure 10:**
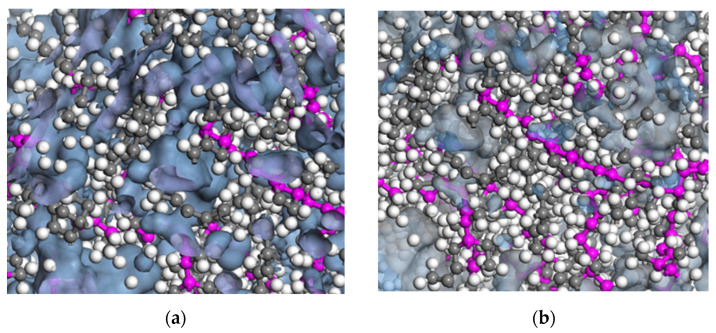
(**a**) Free volume morphology of EPDM at 428 K and (**b**) FFV morphology of EPDM at 478 K.

Figure 10a,b show the morphology of the Connolly volume in the simulation cell; the blue color indicates the accessible volume. The FFV obtained is strongly dependent on the proximity and entanglement between the polymer chains, and it can be seen that the FFV decreases with increasing temperature.

The FFV results obtained for each probe molecule show that the FFV decreases as the interaction attractive force increases. This shortens the distance between the polymer chains, creating crosslinks and entanglement. Consequently, the crosslinking density increases and the FFV decreases with increasing temperature.

The influence of molecular structures on the dynamics of polymers is a central aspect in determining rheological behavior, and properties are known to scale macroscopically. For example, the chain self-diffusion coefficient, D, is mainly determined by the average size and shape of the polymer chain, and the analysis of trajectories such as mean square displacement and radius of gyration allow for elucidating the complex mechanisms of molecular dynamics of rubbery polymers. The diffusion of crosslinked polymers is often described by theories that emphasize the topology of the polymer chains and their assumed motion through a fixed background, representing adjacent chains. However, the complex mechanics of diffusion and crosslinking in amorphous systems can be studied in depth by using molecular dynamics simulations to generate trajectories of the polymer system for more accurate estimation of rheological properties.

In this context, initial studies, such as the one developed by Antonietti and co-workers [43], performed a series of experiments to understand the large-scale viscosity behavior for fluidized polymers in entangled and non-entangled regimes, and found a close relationship of viscosity with the topology of the molecular structure. A significant contribution was the phenomenology of entangled polymers, with the discovery of microgel structures, which confine molecular chains that could not possibly move by reptation.

There is an inherent difficulty in establishing an accurate model for viscosity in fluidized polymers in a wide range of topologies where, in addition, the influence of temperature favors gelling. Years later, Chremos et al. [44] established models to calculate and quantify the size and molecular shapes of fluidized polymers to determine their possible relationship with diffusion and radius of gyration as a complementary measure of the anisotropy of the molecular topology.

The results of Wang et al. [17] establish a physicochemical-specific molecular simulation approach to understand the constitutive behavior of elastomers, and establish a methodology for multiscale analysis relating composition and microstructure to the mechanical performance of rubbery polymers. Wang and co-workers [16] used MD simulations to determine the trajectories, mean square displacement, radius of gyration, and free volume, as well as the self-diffusion behavior and molecular conformation of EPDM rubber, and correlated them with the mechanical behavior.

The results of the molecular dynamics study presented in this paper show that the amount of energy required to induce a conformational change depends on the temperature of the system. According to Saleesung et al. [45], Xie et al. [46], Papanikolaou et al. [19], and Bandyopadhyay et al. [47], as the crosslinking increases and the free volume decreases, the elastic modulus, and thus the tensile strength of the elastomer, increases. This effect is manifested macroscopically as an increase in stiffness or a change in the slope of the stress curve (see Figure 8).

Wang et al. [33] reported that the effect of crosslinking between backbones and physical crosslinking increases the stiffness modulus and decreases the free volume fraction. The results obtained in this study confirm that elevated temperatures weaken molecular interactions, due to the accelerated motion of chain segments, as reflected by changes in the radius of gyration in the segments. This increased mobility eventually leads to the rupture of physical crosslink (see Figure 4).

**Figure 11:**
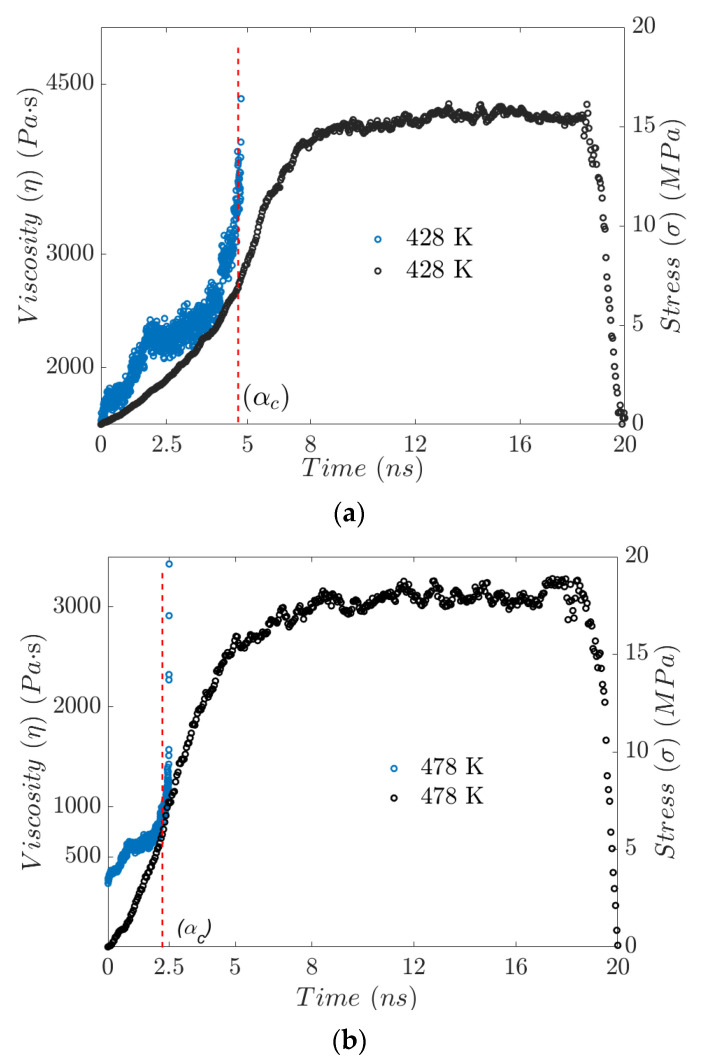
Simulation time dependence on viscosity and stress, (**a**) at 428 K and (**b**) at 478 K.

The dependence of EPDM’s gel point on temperature has been studied from the behavior of the mean squared displacement. As the gel point is approached, the MSD decreases dramatically, to confine the polymer’s molecular network (see Figure 4). EPDM’s intermolecular sliding is restricted as the amount of crosslinking increases, which is manifested by a drastic increase in viscosity. Figure 11a,b show the results of the calculation of stress and viscosity regarding the evolution of the trajectories generated in the molecular dynamics simulations at two different temperatures, 428 and 478 K.

Temperature directly affects the kinetics involved in forming chemical crosslinks. At higher temperatures, crosslink formation is faster, due to an increase in the kinetic energy of the molecules, which increases the likelihood of effective collisions between reactive sites. The greater mobility of the chains at higher temperatures promotes the presence of polar molecules and the formation of weak bonds. Physical crosslinks, therefore, form, which are necessary to reach the gelation point. This process affects the diffusion (mobility and available free volume), causing EPDM chains to have greater elasticity and lower viscosity. Greater chain mobility at elevated temperatures reduces the degree of crosslinking required to form a percolating network, as the chains can more easily rearrange themselves to connect distant sites. Consequently, a lower percentage of crosslinking is necessary at lower temperatures, where the chains are less mobile and require more bonds to form a continuous network.

In other words, the time–temperature relationship for the gel point is inversely proportional. Figure 11a shows that at a temperature of 428 K, it takes approximately 4.9 ns to reach the gel point. Figure 11b shows that the gel point has shifted to the left, with an approximate value of 2.5 ns. The fluidity increases significantly, while the processing time is reduced by 50%.

The physical crosslinking and entanglement of the polymer chains change dynamically, mainly due to temperature. The influence of increased crosslinking and conformational changes is evident in the variation of the resulting viscoelastic properties.

**Figure 12:**
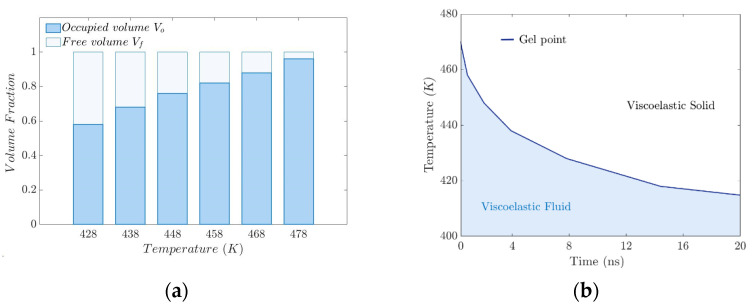
(**a**) Volume fraction distribution versus temperature. (**b**) Critical-phase transition behavior window.

The viscoelastic properties of the rubbery polymer are strongly affected by temperature, and depend on the nature of the interactions between the chains, which can be crosslinking or entanglement. In such a dense set of chains, the strong bonds along the polymer backbone, coupled with the repulsive interaction between unconnected beads, prevent the chains from crossing each other. The result is an entangled system with motion constraints for each chain imposed by entanglement with neighboring chains. Because of the entanglement, the polymers are restricted to repulsive motion. The length, temperature, and stiffness of the chains strongly influence their motion. Figure 12a illustrates the increase in occupied volume referred to in the previous paragraph.

Figure 12b shows the time–temperature window for “moving” the polymer, specifically for the injection process. The intrinsic time of atomistic simulations is infinitesimally short compared to macroscopic time scales. Viscoelastic materials are affected by the size of the system, so the time–temperature superposition principle (TTSP) is proposed, to scale the models. According to TTSP, the mechanical behavior of viscoelastic materials at different temperatures could be related by changing the time domain.

(13) $$S\left(T,t\right)=E\left(T,t/\phi_{t}\right)$$

where S is the target property and $\phi_{t}$ is the transformation factor.

### 3.6. Hardness Test

**Figure 13:**
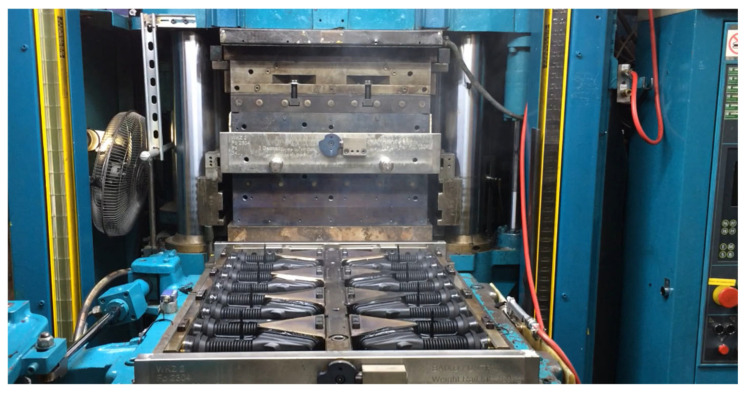
Testing of an injection-molded door grommet for automotive applications.

Six molding tests were performed on an automotive door grommet (see Figure 13), using reference temperatures in molecular dynamics (MD) simulations. Four hardness specimens and tests were obtained using a PCE-DX-AS rubber and elastomer hardness tester (Shore A hardness 0–100+). ASTM D1415-18 and ASTM D2240-15 standards have been used as a point of reference.

**Figure 14:**
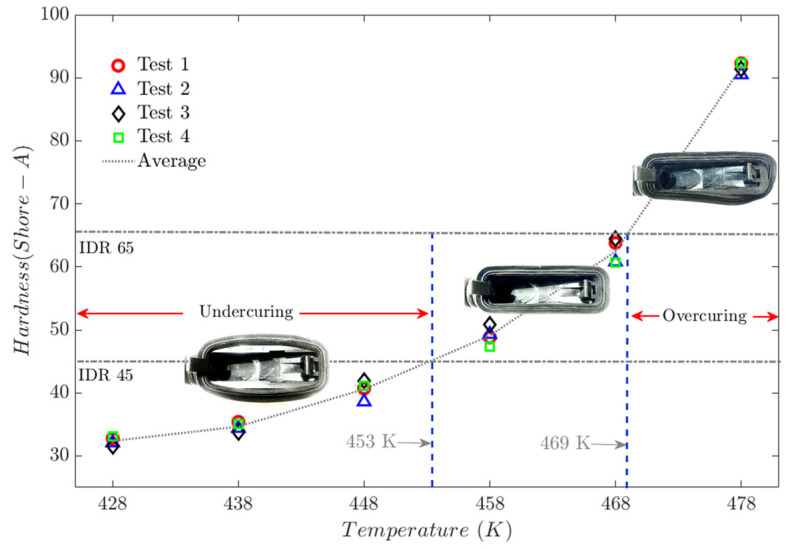
Hardness test on a door grommet application.

The results of the hardness tests are consistent with the MD simulation findings. At temperatures below 453 K, the injected parts exhibit rubbery behavior that tends towards viscosity. In contrast, at temperatures above 469 K, the polymer exhibits rigid properties, with minimal elasticity. Figure 14 shows the hardness test results for a door grommet application.

The International Rubber Hardness Degree (IRHD) test relates to the elasticity of the material [48]. Under this standard, a value between 45 and 65 is considered suitable for door grommet applications.

As discussed in the following paragraphs, the effects of temperature on the macroscopic mechanical properties observed during hardness tests on the door grommet component originate in the conformational structure of the elastomeric polymer at the molecular level.

The density of chemical crosslinking between chains can be considered constant (although the amount of crosslinking depends on the formula of the compound). The time it takes for a compound to reach the limit of this type of crosslinking depends on the catalyzing agents, such as sulfur, and on the temperature during the molding process. Considering only a limited amount of chemical crosslinking, the properties of the elastomers would be similar. The research results of Kumar et al. [49] on the effect of sulfur and carbon black fillers on crosslink density evolution, physical crosslinking, chain mobility, and entanglement evolution are in agreement with the results presented here, corroborating the aforementioned authors’ findings on the intricate interplay between filler-polymer interactions and physical crosslinking dynamics.

The formation of physical crosslinks significantly alters the structural properties. The entanglement of the chains reduces the space available for the backbone to reorganize through reptation, generating greater stiffness. The density of physical crosslinking primarily depends on the curing temperature during the injection process. In their research, Smejda-Krzewicka and Mrozowski [50] confirm the significant effect of crosslinking temperature on the resulting rheological properties.

During the hardness test, the elastomer material undergoes volumetric compaction by the indenter. The extent to which the elastomer can be compacted depends on the void spaces in the polymer matrix, i.e., the free volume fraction.

The above is in line with reports from several researchers that the mechanical properties of elastomeric polymers are significantly affected by the conformational rearrangement of the molecular chains due to crosslinking by weak interactions, entanglements, and the volume fraction occupied by the polymeric networks [49,50,51,52,53].

## 4. Conclusions

Molecular simulation is an effective method for studying and predicting the rheological properties of rubbery polymers and the conformational behavior of molecular chains.

The diffusion phenomenon is highly dependent on the free volume, which is known to be associated with the conformational changes in the backbone and the crosslinking density of the polymer chains.

The evolution of the conformational structure and the internal forces that allow the cohesion of the molecular chains is strongly linked to the temperature; the main conformational changes occur in short periods, so it can be said that the temperature variation is the one that determines the rheological properties for processing and post-processing performance.

The results provide a solid basis for optimizing elastomer injection processes, where balancing initial fluidity and final rigidity is essential. For example, the stabilization of MSD with increasing crosslinking reflects a reduction in chain mobility, consistent with the transition from a molten state to an elastomeric network during vulcanization. Understanding this behavior is crucial for grasping material flow in injection molding and the formation of stable post-vulcanization networks.

The next near-term challenge is multiscale modeling for designing elastomeric components. The development of new modeling tools, including finite-element models, is expected to be stimulated by the increasing use of molecular dynamics simulations to determine mechanical properties from a nanoscopic approach.

In summary, this research emphasizes the potential of molecular dynamics (MD) simulations in guiding the design and processing of elastomers within the rubber injection molding industry. MD simulations offer a predictive tool for optimizing the mechanical and dynamic properties of elastomers. The results pave the way for future research integrating additives and more realistic processing conditions, thereby strengthening the link between simulation and industrial applications. Understanding the processes intrinsic to the dynamics of crosslinking and entanglement of elastomeric polymer chains enables the optimal degree of crosslinking, which in turn allows for improved mechanical properties by designing an efficient curing process during injection molding.

## Figures and Tables

**Table 1:** EPDM compound formulation NORDEL™ IP 4520. in phr (pounds per hundred of rubber).

Substance	Function	Quantity (phr)
Amorphous EPDM	Base elastomer	95–100
Ethylidene norbornene (ENB)	Diene component	2–12
Sulfur	Vulcanizing agent	1.9–2.1
Carbon black (N550 semiactive, specific area ~39–55 m^2^/g)	Promoter	85–95
And other zinc-based processing agents (trade secret).		17–20

## Data Availability

The raw data supporting the conclusions of this article will be made available by the authors on request.

## References

[1] Li S., Tian H., Wu H., Ning N., Tian M., Zhang L. (2020). Coupling effect of molecular weight and crosslinking kinetics on the formation of rubber nanoparticles and their agglomerates in EPDM/PP TPVs during dynamic vulcanization. Soft Matter.

[2] Bouguedad D., Mekhaldi A., Jbara O., Rondot S., Hadjadj A., Douglade J., Dony P. (2015). Physico-chemical study of thermally aged EPDM used in power cables insulation. IEEE Trans. Dielectr. Electr. Insul..

[3] Ravishankar P. (2012). Treatise on EPDM. Rubber Chem. Technol..

[4] Shaji R., Kumar N.N. (2022). Simulation of Room Temperature Vulcanized Gasket Failure at Engine T-Joints. ARAI J. Mobil. Technol..

[5] Restrepo-Zapata N.C., Eagleburger B., Saari T., Osswald T.A., Hernández-Ortiz J.P. (2016). Chemorheological time-temperature-transformation-viscosity diagram: Foamed EPDM rubber compound. J. Appl. Polym. Sci..

[6] Milani G., Milani F. (2011). EPDM accelerated sulfur vulcanization: A kinetic model based on a genetic algorithm. J. Math. Chem..

[7] Niu W., Li Y., Ma Y., Zhao G. (2023). Determination and Prediction of Time-Varying Parameters of Mooney–Rivlin Model of Rubber Material Used in Natural Rubber Bearing under Alternating of Aging and Seawater Erosion. Materials.

[8] Jiang J., Xu J.-s., Zhang Z.-s., Chen X. (2016). Rate-dependent compressive behavior of EPDM insulation: Experimental and constitutive analysis. Mech. Mater..

[9] Habieb A.B., Milani F., Milani G., Pianese G., Torrini D. (2022). Vulcanization degree influence on the mechanical properties of Fiber Reinforced Elastomeric Isolators made with reactivated EPDM. Polym. Test..

[10] Shi C., Cao C., Lei M., Peng L., Shen J. (2015). Time-dependent performance and constitutive model of EPDM rubber gasket used for tunnel segment joints. Tunn. Undergr. Space Technol..

[11] Rashid R.Z.A., Yunus N.A., Mazlan S.A., Johari N., Aziz S.A.A., Nordin N.A., Khairi M.H.A., Johari M.A.F. (2022). Temperature Dependent on Mechanical and Rheological Properties of EPDM-Based Magnetorheological Elastomers Using Silica Nanoparticles. Materials.

[12] Hiranobe C.T., Ribeiro G.D., Torres G.B., Reis E.A.P.d., Cabrera F.C., Job A.E., Paim L.L., Santos R.J.d. (2021). Cross-linked density determination of natural rubber compounds by different analytical techniques. Mater. Res..

[13] Morovati V., Bahrololoumi A., Dargazany R. (2021). Fatigue-induced stress-softening in cross-linked multi-network elastomers: Effect of damage accumulation. Int. J. Plast..

[14] Shen J., Lin X., Liu J., Li X. (2019). Effects of Cross-Link Density and Distribution on Static and Dynamic Properties of Chemically Cross-Linked Polymers. Macromolecules.

[15] Cheng M., Chen W., Song B. (2004). Phenomenological Modeling of the Stress-Stretch Behavior of EPDM Rubber with Loading-rate and Damage Effects. Int. J. Damage Mech..

[16] Wang Y., Liu H., Li P., Wang L. (2022). The Effect of Cross-Linking Type on EPDM Elastomer Dynamics and Mechanical Properties: A Molecular Dynamics Simulation Study. Polymers.

[17] Wang A., Vargas-Lara F., Younker J.M., Iyer K.A., Shull K.R., Keten S. (2021). Quantifying Chemical Composition and Cross-link Effects on EPDM Elastomer Viscoelasticity with Molecular Dynamics. Macromolecules.

[18] Varshney V., Patnaik S.S., Roy A.K., Farmer B.L. (2008). A Molecular Dynamics Study of Epoxy-Based Networks: Cross-Linking Procedure and Prediction of Molecular and Material Properties. Macromolecules.

[19] Papanikolaou M., Drikakis D., Asproulis N. (2015). Molecular dynamics modelling of mechanical properties of polymers for adaptive aerospace structures. AIP Conf. Proc..

[20] van Duin M., Orza R., Peters R., Chechik V. (2010). Mechanism of Peroxide Cross-Linking of EPDM Rubber. Macromol. Symp..

[21] Tsige M., Taylor P.L. (2002). Simulation study of the glass transition temperature in poly(methyl methacrylate). Phys. Rev. E.

[22] Bulacu M., van der Giessen E. (2007). Molecular-dynamics simulation study of the glass transition in amorphous polymers with controlled chain stiffness. Phys. Rev. E.

[23] Duki S., Tsige M., Taylor P. Glass transition temperature of PIB, PDMS and PMMA from small-time simulations. Proceedings of the APS March Meeting Abstracts.

[24] Sun H. (1998). COMPASS:  An ab Initio Force-Field Optimized for Condensed-Phase ApplicationsOverview with Details on Alkane and Benzene Compounds. J. Phys. Chem. B.

[25] McQuaid M.J., Sun H., Rigby D. (2004). Development and validation of COMPASS force field parameters for molecules with aliphatic azide chains. J. Comput. Chem..

[26] Saha S., Bhowmick A.K. (2019). An Insight into molecular structure and properties of flexible amorphous polymers: A molecular dynamics simulation approach. J. Appl. Polym. Sci..

[27] Guo Y., Liu J., Lu Y., Dong D., Wang W., Zhang L. (2018). A combined molecular dynamics simulation and experimental method to study the compatibility between elastomers and resins. Rsc Adv..

[28] Yang W., Chen X., Song X., Hu Y., Pei J., Chen J. (2024). Molecular dynamics study on the physical compatibility of SEBS/plasticizer blend systems. J. Mol. Model..

[29] Xiang Z., Gao C., Long T., Ding L., Zhou T., Wu Z. (2025). Force-Field Benchmark for Polydimethylsiloxane: Density, Heat Capacity, Isothermal Compressibility, Viscosity and Thermal Conductivity. J. Phys. Chem. B.

[30] Odegard G.M., Patil S.U., Deshpande P.P., Kanhaiya K., Winetrout J.J., Heinz H., Shah S.P., Maiaru M. (2021). Molecular dynamics modeling of epoxy resins using the reactive interface force field. Macromolecules.

[31] Joseph E., Swaminathan N., Kannan K. (2020). Material identification for improving the strength of silica/SBR interface using MD simulations. J. Mol. Model..

[32] Yu R., Wang Q., Wang W., Xiao Y., Wang Z., Zhou X., Zhang X., Zhu X., Fang C. (2021). Polyurethane/graphene oxide nanocomposite and its modified asphalt binder: Preparation, properties and molecular dynamics simulation. Mater. Des..

[33] Wang Y., Yang Y., Tao M. (2019). Understanding Free Volume Characteristics of Ethylene-Propylene-Diene Monomer (EPDM) through Molecular Dynamics Simulations. Materials.

[34] Zachary M., Camara S., Whitwood A.C., Gilbert B.C., van Duin M., Meier R.J., Chechik V. (2008). EPR study of persistent free radicals in cross-linked EPDM rubbers. Eur. Polym. J..

[35] Paterlini M.G., Ferguson D.M. (1998). Constant temperature simulations using the Langevin equation with velocity Verlet integration. Chem. Phys..

[36] Yin K., Xiao H., Zhong J., Xu D. A new method for Calculation of Elastic Properties of Anisotropic material by constant pressure molecular dynamics. Proceedings of the International Conference of Computational Methods in Sciences and Engineering 2004 (ICCMSE 2004).

[37] Varadwaj P.R. (2020). Combined Molecular Dynamics and DFT Simulation Study of the Molecular and Polymer Properties of a Catechol-Based Cyclic Oligomer of Polyether Ether Ketone. Polymers.

[38] Gómez-Jimenez S., Saucedo-Anaya T., Baltazar-Hernandez V.H., Contreras-Rodriguez A.R. (2023). Characterization of Viscoelastic Properties of EPDM Molding Compound for Door Grommet Component Using Molecular Dynamics and Phenomenological Modeling. J. Manuf. Sci. Eng..

[39] (2019). Standard Test Method for Rubber Property—Vulcanization Using Oscillating Disk.

[40] Sun X., Isayev A.I. (2009). Cure Kinetics Study of Unfilled and Carbon Black Filled Synthetic Isoprene Rubber. Rubber Chem. Technol..

[41] Milani G., Leroy E., Milani F., Deterre R. (2013). Mechanistic modeling of reversion phenomenon in sulphur cured natural rubber vulcanization kinetics. Polym. Test..

[42] Gómez-Jiménez S., Saucedo-Anaya T., López-Baltazar E.A., Robles-Guerrero A. (2024). Automated identification of kinetics model in elastomer vulcanization using a rheometer MDR and Rubber-K pattern recognition software. Results Mater..

[43] Antonietti M., Pakula T., Bremser W. (1995). Rheology of Small Spherical Polystyrene Microgels: A Direct Proof for a New Transport Mechanism in Bulk Polymers besides Reptation. Macromolecules.

[44] Chremos A., Jeong C., Douglas J.F. (2017). Influence of polymer architectures on diffusion in unentangled polymer melts. Soft Matter.

[45] Saleesung T., Reichert D., Saalwächter K., Sirisinha C. (2015). Correlation of crosslink densities using solid state NMR and conventional techniques in peroxide-crosslinked EPDM rubber. Polymer.

[46] Xie B.-G., Wang H., Lu R.-L., Wang H., Xia R., Chen P., Qian J.-S. (2020). A combined simulation and experiment study on polyisoprene rubber composites. Compos. Sci. Technol..

[47] Bandyopadhyay A., Valavala P.K., Clancy T.C., Wise K.E., Odegard G.M. (2011). Molecular modeling of crosslinked epoxy polymers: The effect of crosslink density on thermomechanical properties. Polymer.

[48] (2018). Standard Test Method for Rubber Property—International Hardness.

[49] P P., Neethirajan J., M.I. K., R R., R R. (2025). Thermo-oxidative aging and its influence on the performance of silica, carbon black, and silica/carbon black hybrid fillers -filled tire tread compounds. J. Polym. Res..

[50] Smejda-Krzewicka A., Mrozowski K. (2025). Chloroprene and Butadiene Rubber (CR/BR) Blends Cross-Linked with Metal Oxides: INFLUENCE of Vulcanization Temperature on Their Rheological, Mechanical, and Thermal Properties. Molecules.

[51] Gomez-Jimenez S., Saucedo-Anaya T., Guerrero-Mendez C., Robles-Guerrero A., Silva-Acosta L., Navarro-Solis D., Lopez-Betancur D., Contreras Rodríguez A.R. (2024). Mooney–Rivlin Parameter Determination Model as a Function of Temperature in Vulcanized Rubber Based on Molecular Dynamics Simulations. Materials.

[52] Shao H., Guo Q., He A. (2022). Evolution of crosslinking networks structure and thermo-oxidative aging behavior of unfilled NR/BR blends with TBIR as extra functional compatibilizer. Polym. Test..

[53] Panyukov S. (2020). Theory of Flexible Polymer Networks: Elasticity and Heterogeneities. Polymers.

